# Discovering Breast Cancer Biomarkers Candidates through mRNA Expression Analysis Based on The Cancer Genome Atlas Database

**DOI:** 10.3390/jpm12101753

**Published:** 2022-10-21

**Authors:** Dong Hyeok Kim, Kyung Eun Lee

**Affiliations:** 1 Department of Clinical Laboratory Science, College of Health Sciences, Catholic University of Pusan, Busan 46252, Korea; 2 Clinical Trial Specialist Program for In Vitro Diagnostics, Brain Busan 21 Plus Program, The Graduate School, Catholic University of Pusan, Busan 46252, Korea

**Keywords:** biomarker, mRNA, breast cancer, The Cancer Genome Atlas

## Abstract

Background: Research on the discovery of tumor biomarkers based on big data analysis is actively being conducted. This study aimed to secure foundational data for identifying new biomarkers of breast cancer via breast cancer datasets in The Cancer Genome Atlas (TCGA). Methods: The mRNA profiles of 526 breast cancer and 60 adjacent non-cancerous breast tissues collected from TCGA datasets were analyzed via MultiExperiment Viewer and GraphPad Prism. Diagnostic performance was analyzed by identifying the pathological grades of the selected differentially expressed (DE) mRNAs and the expression patterns of molecular subtypes. Results: Via DE mRNA profile analysis, we selected 14 mRNAs with downregulated expression (HADH, CPN2, ADAM33, TDRD10, SNF1LK2, HBA2, KCNIP2, EPB42, PYGM, CEP68, ING3, EMCN, SYF2, and DTWD1) and six mRNAs with upregulated expression (ZNF8, TOMM40, EVPL, EPN3, AP1M2, and SPINT2) in breast cancer tissues compared to that in non-cancerous tissues (*p* < 0.001). Conclusions: In total, 20 DE mRNAs had an area under cover of 0.9 or higher, demonstrating excellent diagnostic performance in breast cancer. Therefore, the results of this study will provide foundational data for planning preliminary studies to identify new tumor biomarkers.

## 1. Introduction

At present, the representative tumor markers used for breast cancer diagnosis in clinical practice are cancer antigen 15-3 (CA15-3) and carcinoembryonic antigen (CEA); these are approved for breast cancer monitoring by the United States Food and Drug Administration [1,2,3]. CA15-3 can be used to monitor patients during treatment, predict tumor recurrence, and monitor the treatment of patients with metastatic breast cancer [4]. CEA, a carcinogenic protein, is a positive marker for several cancers such as colorectal, pancreatic, stomach, breast, and lung cancers [5]. However, it is known that different countries and institutions have different opinions regarding CA15-3 and CEA. The European Group on Tumor Markers recommends the use of CA15-3 and CEA for the early detection of disease, treatment monitoring, and prognosis evaluation [6]. The National Comprehensive Cancer Network guidelines do not recommend using CA15-3 and CEA as markers for pretreatment and clinical evaluation [7]. Further, the American Society of Clinical Oncology guidelines do not recommend the use of CA15-3 and CEA for diagnosis, staging, and therapeutic monitoring [8]. Although opinions on the tumor markers for breast cancer currently used in clinical practice are divided, alternative proposals for new tumor markers for breast cancer are not accurately presented. Therefore, the discovery of critical biomarkers that will aid in early diagnosis, treatment, and prognosis is considered very important in breast cancer research.

Recently, research on the discovery of tumor biomarkers based on data analysis of The Cancer Genome Atlas (TCGA) is actively being conducted. TCGA is a cancer-specific multi-omics data resource, and it is known that 33 types of cancer data obtained from approximately 11,300 patients comprise the vast amount of data of approximately 2 petabytes [9,10,11]. The purpose of this TCGA project was to comprehensively characterize the molecular events of primary cancer and make them available to all researchers [12]. Therefore, TCGA data will be used as practical data to support and analyze research by numerous researchers.

In a study based on TCGA data, it was reported that transcriptional repressor GATA binding 1 was specifically differentially expressed (DE) only in triple-negative breast cancer (TNBC), and that the RNA binding motif single stranded interacting protein 3 was downregulated when the prognosis of breast cancer patients was poor [13,14]. In addition, many research results have been published in tumor research other than that of breast cancer. In the case of cholangiocarcinoma, the cluster of differentiation 247, Fc gamma receptor Ia, and transformation/transcription-domain-associated proteins were suggested as new markers for mRNA-based cancer vaccine targets, and significance with a high expression of the family with sequence similarity 65 member A was also reported in colorectal cancer [15,16]. Generally, TCGA data-based analysis is considered to increase biological insight by studying the tumor environment based on a vast amount of analysis.

Therefore, in this study, the mRNA expression data of 526 breast cancers and 60 adjacent non-cancerous breast tissues stored in TCGA were analyzed to identify new biomarkers for breast cancer. The diagnostic significance of new breast cancer biomarkers was confirmed via receiver operating characteristic (ROC) curve analysis of DE mRNAs and the area under the curve (AUC) value analysis of sensitivity and specificity, and these were intended to be used as basic data for discovering new breast cancer biomarker candidates.

## 2. Materials and Methods

### 2.1. Public Data Collection

Datasets for mRNA expression data and clinical data of breast cancer were obtained from Firebrowse “http://firebrowse.org (accessed on 13 October 2019)”. Firebrowse is a user-friendly interface for analyzing reports generated via the Broad Institute’s TCGA-GDAC firehose pipeline containing processed TCGA data [17]. To identify DE mRNAs and determine their clinical diagnostic performance, data for all clinical samples, including age, race, tumor stage, molecular subtype, and expression level (log2 normalization), were included for 526 breast cancer and 60 adjacent non-cancerous breast tissues. Sixty adjacent non-cancerous breast tissues were paired with 60 of the 526 breast cancer samples.

### 2.2. mRNA Expression Analysis

To obtain the profiles of DE mRNAs in breast cancer, we investigated TCGA data. The intersection of data from 60 breast cancer samples paired with the data of 60 adjacent non-cancerous breast tissues and 526 breast cancer with the data of 60 adjacent non-cancerous breast tissues were selected to identify significant mRNA via volcano plot analyses using the MultiExperiment Viewer (MeV 4.4; The Perl Foundation, Holland, MI, USA) from 17,815 mRNAs.

ROC curve analysis and the AUC were analyzed using GraphPad Prism software version 8 (La Jolla, CA, USA) to determine the differential expression level of the selected mRNAs. To select mRNAs with a substantial diagnostic performance, mRNAs with an AUC value of 0.9 or higher were selected and analyzed through hierarchical clustering.

Heatmap analysis and hierarchical clustering to confirm the expression pattern of the selected DE mRNAs were performed using MEV software version 4.4 to determine the mRNA expression profile and identify DE mRNA.

### 2.3. Diagnostic Performance Analysis

To examine the diagnostic performance of the selected DE mRNAs, expression patterns were analyzed at each stage in the tumor stages, divided into stages I, II, III, and IV; molecular subtypes were divided into Luminal A (estrogen receptor (ER) or progesterone receptor (PgR) positive, human epidermal growth factor receptor type 2 (HER2) negative), Luminal B (ER- or PgR-positive, HER2-positive), HER2-positive, and TNBC.

### 2.4. Statistical Analysis

Statistical analyses were performed using MEV version 4.4 and GraphPad Prism version 8 (La Jolla, CA, USA). Student’s *t*-test or one-way analysis of variance (ANOVA) was used to compare mRNA expression, tumor stages, and molecular subtypes between cancerous and non-cancerous breast tissues. In all analyses, the differences were considered statistically significant when *p* < 0.05 (* *p* < 0.05, ** *p* < 0.01, *** *p* < 0.001).

## 3. Results

### 3.1. Characteristics of Breast Cancer Patients

The mRNA dataset consisting of 526 breast cancer and 60 adjacent non-cancerous breast tissues utilized in this study was obtained from TCGA. Table 1 summarizes the clinical data of breast cancer patients. Among the 526 total breast cancer cases, the proportion of female patients was 98.86% (520/526). The age of the patients was 57.91 ± 13.26 years. The most frequently occurring race was Caucasian (68.63%, 361/526), followed by unknown (17.11%, 90/526), African American (7.60%, 40/526), Asian (6.46%, 34/526), and American Indian or Alaskan native (0.19%, 1/526). The tumor stages of the patients were 16.92% (89/526) for Stage I, 56.27% (296/526) for Stage II, 20.91% (110/526) for Stage III, 2.47% (13/526) for Stage IV, and 3.43% (18/526) for Unknown. The molecular subtypes of the patients were 62.74% (330/526) for Luminal A, 15.02% (79/526) for Luminal B, 4.75% (25/526) for HER2-positive cancer, and 17.49% (92/526) for TNBC.

### 3.2. Differential Expression of Breast Cancer mRNA Profiles

**Figure 1:**
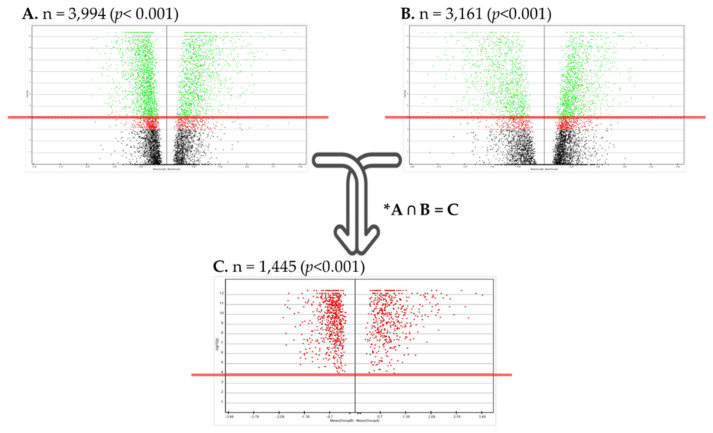
Volcano spots for screening differentially expressed mRNAs. Cut-off point was −log 10(*p*) > 3. (**A**) 526 breast cancer and 60 adjacent non-cancerous breast tissues, (**B**) 60 breast cancer paired with 60 adjacent non-cancerous breast tissues, (**C**) intersection of (**A**,**B**), * (**A**) ∩ (**B**) = (**C**); Intersection (**A**,**B**).

DE mRNAs profiles were investigated in breast cancer using MEV version 4.4. DE mRNAs containing significant intersections in 526 breast cancer and 60 non-adjacent breast tissues, and 60 breast cancer paired with 60 non-adjacent breast tissues, and were selected based on −log10(*p*) > 3 from a total of 17,815 mRNAs. Of the 17,815 mRNAs, 1,445 mRNAs were found to form an intersection (Figure 1).

The diagnostic performance of 1,445 selected DE mRNAs was determined using ROC curve analysis. 20 DE mRNAs (*p* < 0.0001) with an area under the curve (AUC) value of 0.9 or higher were selected. Results for selected DE mRNAs are listed in Table 2. The mRNA with the highest AUC value was ING3 (0.96, 95% CI = 0.94–0.97), and the mRNA with the lowest AUC value was SPINT2 (0.90, 95% difference = 0.87–0.93).

**Figure 2:**
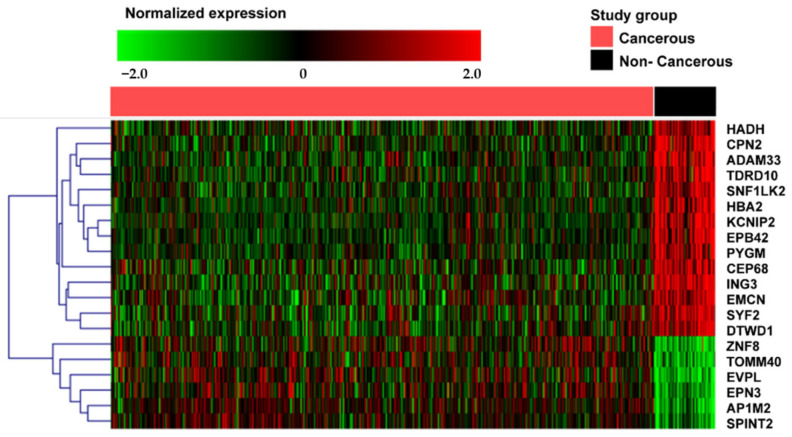
Heatmap of selected 20 mRNAs from cancerous and non-cancerous tissues. Heatmap is obtained using bidirectional hierarchical clustering of 20 significantly expressed mRNAs (*p* < 0.05 by Pearson correlation, hierarchical clustering analysis). Red dots indicate upregulated mRNAs and green dots indicate downregulated mRNAs.

In addition, the 20 selected DE mRNAs were investigated for trends in breast cancer through a heatmap. Of the 20 DE mRNAs, the expressions of 14 DE mRNAs (HADH, CPN2, ADAM33, TDRD10, SNF1LK2, HBA2, KCNIP2, EPB42, PYGM, CEP68, ING3, EMCN, SYF2, and DTWD1) were downregulated in cancerous tissues compared to that in non-cancerous tissues, and the expressions of six DE mRNAs (ZNF8, TOMM40, EVPL, EPN3, AP1M2, and SPINT2) were upregulated in cancerous tissues compared to that in non-cancerous tissues (Figure 2).

### 3.3. Diagnostic Performance Analysis via mRNA Expression Profile Verification

**Figure 3:**
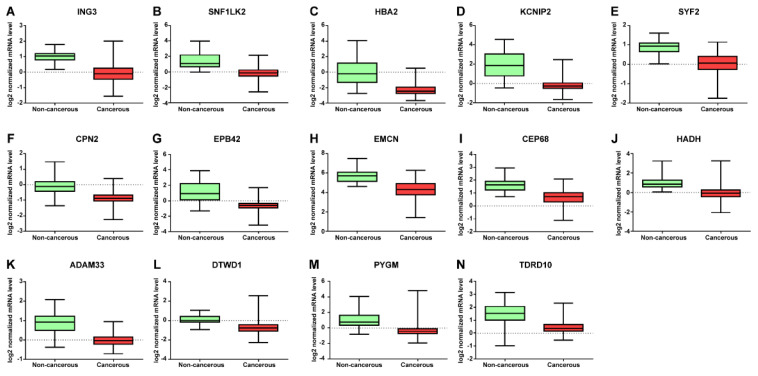
Expression levels of downregulated mRNA in breast cancer tissues. 14 differentially expressed (DE) mRNA profile data with *p* < 0.001 values. (**A**) ING3, (**B**) SNF1LK2, (**C**) HBA2, (**D**) KCNIP2, (**E**) SYF2, (**F**) CPN2, (**G**) EPB42, (**H**) EMCN, (**I**) CEP68, (**J**) HADH, (**K**) ADAM33, (**L**) DTWD1, (**M**) PYGM, and (**N**) TDRD10.

**Figure 4:**
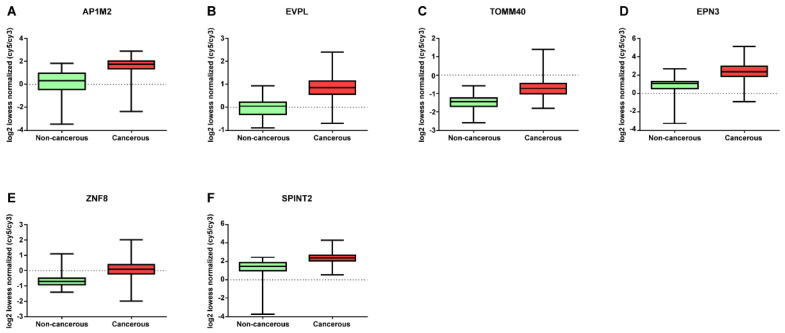
Expression levels of upregulated mRNA in non-cancerous tissues. 6 DE mRNA profile data with *p* < 0.001 values. (**A**) AP1M2, (**B**) EVPL, (**C**) TOMM40, (**D**) EPN3, (**E**) ZNF8, and (**F**) SPINT2.

mRNA expression profile was verified to investigate its diagnostic performance. It was found to be lower (HADH, CPN2, ADAM33, TDRD10, SNF1LK2, HBA2, KCNIP2, EPB42, PYGM, CEP68, ING3, EMCN, SYF2, and DTWD1) or higher (ZNF8, TOMM40, EVPL, EPN3, AP1M2, and SPINT2) in breast cancer tissue than in adjacent non-cancerous tissue (Figure 3 and Figure 4).

**Figure 5:**
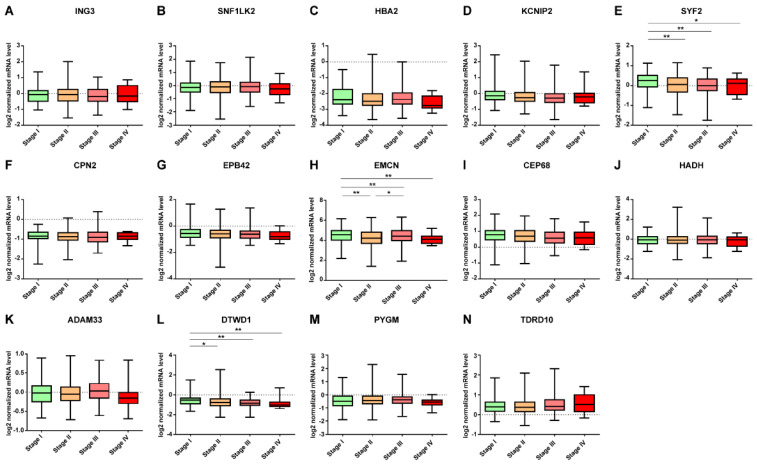
Expression patterns of DE mRNAs downregulated in breast cancer tissue according to tumor stages. (**A**) ING3, (**B**) SNF1LK2, (**C**) HBA2, (**D**) KCNIP2, (**E**) SYF2, (**F**) CPN2, (**G**) EPB42, (**H**) EMCN, (**I**) CEP68, (**J**) HADH, (**K**) ADAM33, (**L**) DTWD1, (**M**) PYGM, and (**N**) TDRD10. Data are reported as mean ± SD. * *p* < 0.05, ** *p* < 0.01.

**Figure 6:**
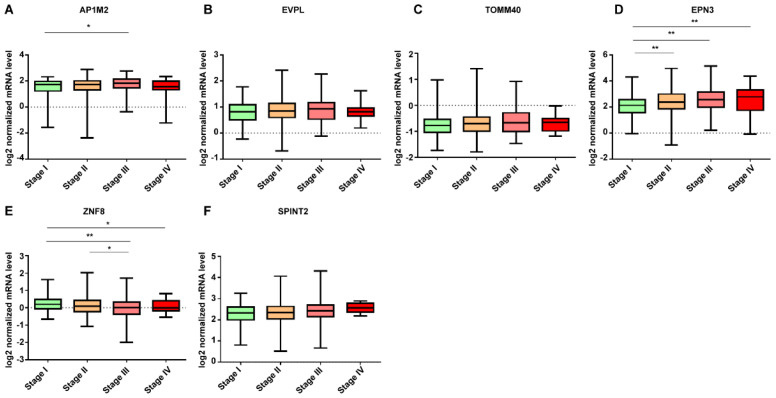
Expression patterns of DE mRNAs upregulated in breast cancer tissue according to tumor stages. (**A**) AP1M2, (**B**) EVPL, (**C**) TOMM40, (**D**) EPN3, (**E**) ZNF8, and (**F**) SPINT2. Data are reported as mean ± SD. * *p* < 0.05, ** *p* < 0.01.

The expression pattern of DE mRNA according to the tumor stage in mRNAs was downregulated in breast cancer tissue, SYF2 and DTWD1 showed a more down-expressed pattern as the cancer progressed (Figure 5). In upregulated mRNAs in breast cancer tissue, EPN3 was up-expressed as the cancer progressed, and ZNF8 was down-expressed as the cancer progressed (Figure 6). Data from 18 samples were excluded from this study because the stage was unknown.

**Figure 7:**
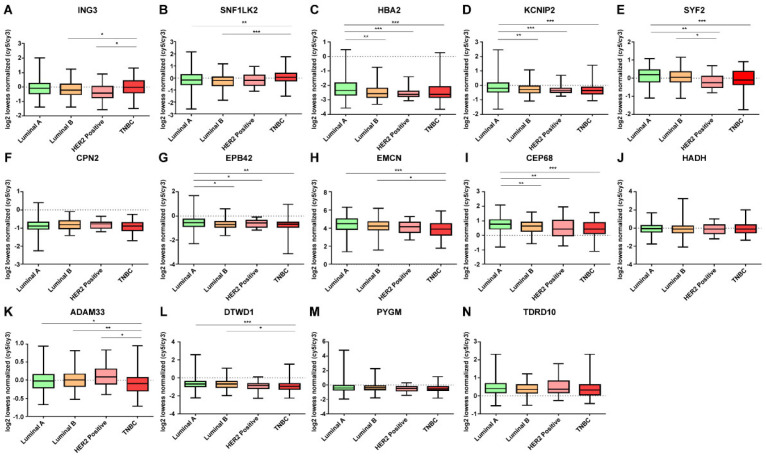
Expression patterns of DE mRNAs downregulated in breast cancer tissue according to molecular subtypes. (**A**) ING3, (**B**) SNF1LK2, (**C**) HBA2, (**D**) KCNIP2, (**E**) SYF2, (**F**) CPN2, (**G**) EPB42, (**H**) EMCN, (**I**) CEP68, (**J**) HADH, (**K**) ADAM33, (**L**) DTWD1, (**M**) PYGM, and (**N**) TDRD10. Data are reported as mean ± SD. * *p* < 0.05, ** *p* < 0.01, *** *p* < 0.001.

**Figure 8:**
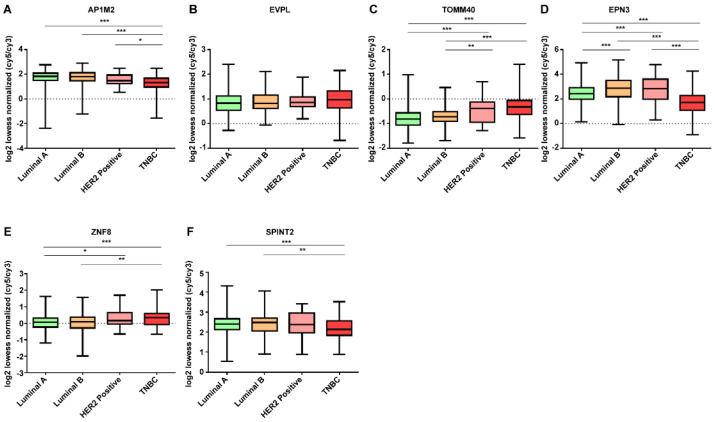
Expression patterns of DE mRNAs upregulated in breast cancer tissue according to molecular subtypes. (**A**) AP1M2, (**B**) EVPL, (**C**) TOMM40, (**D**) EPN3, (**E**) ZNF8, and (**F**) SPINT2. Data are reported as mean ± SD. * *p* < 0.05, ** *p* < 0.01, *** *p* < 0.001.

In addition, we investigated whether DE mRNAs whose expression were downregulated and upregulated in breast cancer were correlated with molecular subtype. The expression levels of DE mRNAs according to molecular subtype status are shown in Figure 7 and Figure 8. DE mRNAs downregulated in breast cancer tissue were not significant in molecular subtypes. However, among the DE mRNAs whose expressions were upregulated in breast cancer tissues, TOMM40 was highly expressed in TNBC compared to that seen in other molecular subtypes, and EPN3 was expressed lower in TNBC than in other molecular subtypes.

## 4. Discussion

Recently, research on the discovery of tumor biomarkers based on big data analysis is actively being conducted, such as the use of TCGA data to identify these biomarkers [13,14,15,16]. Online bioinformatics tools contain big data that provide comprehensive genetic information for various cancers via microarray and next-generation sequencing technology [10,11]. These online bioinformatics tools have provided numerous researchers with data to publish new research papers, resulting in increased biological insight. Therefore, research on the discovery of new biomarkers related to tumor development is actively being conducted in clinical practice, and breast cancer is no exception. CA15-3 and CEA tumor markers are being used to diagnose, treat, and predict breast cancer; however, as limitations such as the low efficacy and low sensitivity of early diagnosis are revealed, expectations for new tumor markers to supplement these in the future are increasing [18,19,20]. Therefore, in this study, DE mRNAs were identified by analyzing the mRNA expression data of 526 breast cancer and 60 adjacent non-breast cancer tissues collected from TCGA data and, based on this, an attempt was made to secure preliminary data to discover new biomarkers that would aid in the diagnosis and treatment breast cancer.

Since TCGA data used in this study were collected from non-cancerous tissue data of patients such as breast cancer tissue, a primary screening analysis was attempted after considering these clinical characteristics. Through volcano spot analysis, significant 3994 mRNAs were identified in 526 breast cancer tissues and 60 adjacent non-mammary tissues, and significant 3161 mRNAs were identified in 60 adjacent non-mammary cancer tissues of the patient, such as 60 breast cancer tissue samples. Then, a significantly (*p* < 0.001) number of 1445 mRNAs in both groups were selected and this study was performed.

Through ROC analysis of 1,445 mRNAs selected via volcano spot analysis, a total of 20 mRNAs (ING3, SNF1LK2, EVPL, HBA2, KCNIP2, SYF2, AP1M2, CPN2, EPB42, TOMM40, EMCN, CEP68, HADH, ADAM33, EPN3, ZNF8, DTWD1, PYGM, TDRD10, SPINT2) with an AUC value of 0.9 or higher were identified (Table 2). AUC values through ROC analysis indicate diagnostic accuracy and are classified as AUC = 0.5 (non-informative), 0.5 < AUC ≤ 0.7 (less accurate), 0.7 < AUC ≤ 0.9 (moderately accurate), 0.9 < AUC ≤ 1.0 (highly accurate) and AUC = 1 (perfect) [21]. Therefore, the 20 mRNAs identified in this study are highly likely to serve as new biomarkers with a highly accurate AUC value (0.9 < AUC ≤ 1.0). Through a heat map analysis of the 20 selected DE mRNAs, 14 mRNAs (HADH, CPN2, ADAM33, TDRD10, SNF1LK2, HBA2, KCNIP2, EPB42, PYGM, CEP68, ING3, EMCN, SYF2, DTWD1) were found to be downregulated in breast cancer tissues and six mRNAs (ZNF8, TOMM40, EVPL, EPN3, AP1M2, SPINT2) were upregulated in breast cancer tissues (Figure 2).

The expression patterns of the 20 DE mRNAs identified in this study were compared with those reported in other papers published from 2000 to 2022 that studied the expression patterns of various tumors including breast cancer (Table 3). There were nine mRNAs (ING3, SNF1LK2, SYF2, CPN2, EMCN, ADAM33, TDRD10, EPN3, SPINT2) for which there was at least one or more study related to breast cancer. Eleven mRNAs (ING3, SNF1LK2, SYF2, CPN2, EPB42, EMCN, HADH, DTWD1, PYGM, EPN3, SPINT2) with at least one or more other tumor-related study were found. The expression patterns of SYF2 and CPN2 in other breast cancer studies and in studies of other tumor types were both opposite to the findings of this study [22,23,24,25]. In addition, EPN3 was upregulated in other breast cancer-related studies, similar to this study, but was downregulated in gastric cancer and upregulated in glioblastoma, with the opposite results [26,27,28]. SPINT2 showed various expression patterns in tumors. In other studies related to breast cancer, results that contradicted the results of this study were reported, and other tumor-related studies (liver, renal, gastric, cervical, prostate cancer, and medulloblastoma) showed that SPINT2 expression was downregulated [29,30,31,32]. However, studies related to other tumors, including breast cancer, were insufficient overall. Therefore, if a prospective study of 20 DE mRNAs selected based on the analysis results of this study is performed on breast cancer patients, it is expected that a new biomarker can be specifically identified.

In this study, as the pathological stage increased, SYF2, DTWD1, and ZNF8 were downregulated, and EPN3 was upregulated (Figure 5 and Figure 6). First, SYF2 in showed significant differences in pathological stages stage I-IV (*p* < 0.05), stage I-III (*p* < 0.01), and stage I-II (*p* < 0.01), As the pathological stage progressed, the expression level decreased. However, in other studies, SYF2 expression was upregulated in cancer tissues compared to that in normal tissues as the tumor grade increased in ovarian and breast cancers [10,23]. DTWD1 showed a significant difference in the stage I-IV (*p* < 0.01), stage I-III (*p* < 0.01), stage I-II (*p* < 0.05), and the expression level decreased as the pathological stage progressed. However, there are very few studies on the pathological stage in other tumors, including breast cancer, making it difficult to draw comparisons. In addition, ZNF8 showed significant differences in stages I–IV (*p* < 0.05), stages I–III (*p* < 0.01), and stages II–III (*p* < 0.05), and its expression was upregulated in breast cancer tissues compared to that in normal tissues: the higher the pathological stage, the more its expression was downregulated. However, there have been very few tumor-related studies on ZNF8, and its function is not clearly known. In this study, EPN3 showed significant differences in stage I–IV (*p* < 0.01), stage I–III (*p* < 0.01), and stage I–II (*p* < 0.01) and the higher the pathological stage, the higher the expression level. EPN3 expression has been reported to be upregulated in high-grade tissues compared to that in low-grade tissues in glioblastoma [27]. In addition, EPN3 has been reported to enhance the migration and invasion of cancer cells [53]. However, EPN3 expression patterns could not be compared because there are very few studies related to breast cancer. The pathological stage is one of the major determinants influencing the decision to use systemic therapy for breast cancer patients [54]. Therefore, SYF2, DTWD1, ZNF8, and EPN3 are biomarkers that can help to differentiate pathological stages and are judged to be helpful in determining the appropriate treatment for patients.

According to receptor status, molecular subtypes are broadly classified into four types, Luminal A (ER- or PgR- positive, HER2-negative), Luminal B (ER- or PgR-positive, HER2-positive), HER2-positive and TNBC, and are known to be key factors in determining the treatment strategy in early breast cancer patients [55,56]. Therefore, it is important that the discovery of biomarkers that help to distinguish molecular subtypes will aid in determining the appropriate treatment method for patients. While classifying molecular subtypes in this study, TOMM40 and EPN3 in Figure 8 showed significant results. TOMM40 showed a significant difference with *p* < 0.001 in most molecular subtypes: It was found to be highly expressed in TNBC. EPN3 showed a significant difference with *p* < 0.001 in all types except for HER2-positive molecular subtype Luminal B and HER2-positive. EPN3 is highly expressed in HER2 receptor-expressing molecular subtypes (Luminal B, HER2-positive), and its expression is downregulated in TNBC, which is considered to be significant in distinguishing molecular subtypes. TNBC is known to have a poor prognosis compared to other molecular subtypes of breast cancer due to its high heterogeneity, aggression, and lack of treatment options [57,58]. Therefore, TOMM40 and EPN3 are expected to serve as screening factors for differentiating TNBC and other molecular subtypes.

## 5. Conclusions

In conclusion, 20 DE mRNAs with high diagnostic accuracy with an AUC value > 0.9 or higher were identified via TCGA data analysis, and their diagnostic performance was confirmed. This would aid in the discovery of new tumor markers for breast cancer. Among the 20 identified DE mRNAs, EPN3 expression was upregulated in breast cancer tissues compared to that in normal tissues and was upregulated as the histopathological grade of breast cancer increased. In addition, the expression pattern of EPN3 varied depending on the expression of the receptor; therefore, EPN3 was considered to have high diagnostic value. This can be expected to serve as a helpful marker when diagnosing breast cancer and deciding on the direction of treatment. However, as in a study of TCGA data analysis, it was not possible to plan an even distribution with respect to the type and number of samples and clinical characteristics. Hence, validation experiments were been conducted on the DE mRNAs identified at the clinical sample, cellular, or animal levels. Therefore, it is expected that the results of data analysis from this study will serve as basic data when planning preliminary studies to discover new tumor markers in the future.

## Figures and Tables

**Table 1:** Clinical data of enrolled individuals in the current study.

Characteristics	Breast Cancer, *n* = 526 *n*, (%)
Female	520, (98.86)
Age (y, mean ± SD)	57.91, ± 13.26
Race	
Asian	34, (6.46)
African American	40, (7.60)
Caucasian	361, (68.63)
American Indian or Alaska native	1, (0.19)
Unknown	90, (17.11)
Tumor stage	
Stage I	89, (16.92)
Stage II	296, (56.27)
Stage III	110, (20.91)
Stage IV	13, (2.47)
Unknown	18, (3.43)
Molecular subtype	
Luminal A	330, (62.74)
Luminal B	79, (15.02)
HER2-positive	25, (4.75)
TNBC	92, (17.49)

**Table 2:** Clinical diagnostic performance of 20 mRNAs differentially expressed in breast cancer.

mRNAs	Log2 Normalized mRNA Level		AUC	Cutoff	Sensitivity	Specificity	*p*-Value
	Non-Cancerous	Cancerous					
ING3	0.9884 ± 0.03998	−0.09470 ± 0.2354	0.96 (0.94–0.97)	<0.5343	87.71% (84.61–90.39)	91.67% (81.61–97.24)	<0.0001
SNF1LK2	1.426 ± 0.1338	−0.1613 ± 0.02701	0.94 (0.92–0.97)	<0.4393	85.82% (82.56–88.68)	88.33% (77.43–95.18)	<0.0001
EVPL	−0.02857 ± 0.08834	0.8723 ± 0.01995	0.94 (0.92–0.97)	>0.4566	84.12% (80.72–87.13)	93.33% (83.80–98.15)	<0.0001
HBA2	0.06397 ± 0.2116	−2.279 ± 0.02886	0.93 (0.90–0.97)	<−1.616	85.07% (81.74–88.00)	86.67% (75.41–94.06)	<0.0001
KCNIP2	1.955 ± 0.1823	−0.1219 ± 0.02623	0.93 (0.90–0.96)	<0.3937	85.82% (82.56–88.68)	86.67% (75.41–94.06)	<0.0001
SYF2	0.8908 ± 0.07179	0.06124 ± 0.02065	0.93 (0.90–0.96)	<0.5191	82.99% (79.51–86.09)	90.00% (79.49–96.24)	<0.0001
AP1M2	−0.04386 ± 0.1699	1.649 ± 0.02720	0.93 (0.90–0.96)	>1.111	86.39% (83.17–89.20)	86.67% (75.41–94.06)	<0.0001
CPN2	−0.08433 ± 0.08377	−0.8673 ± 0.01309	0.93 (0.89–0.97)	<−0.5644	85.44% (82.15–88.34)	88.33% (77.43–95.18)	<0.0001
EPB42	1.208 ± 0.1597	−0.5775 ± 0.02256	0.93 (0.89–0.97)	<−0.2037	82.99% (79.51–86.09)	86.67% (75.41–94.06)	<0.0001
TOMM40	−1.474 ± 0.07094	−0.6728 ± 0.01989	0.92 (0.89–0.96)	>−1.093	82.04% (78.50–85.22)	86.67% (75.41–94.06)	<0.0001
EMCN	5.715 ± 0.1217	4.270 ± 0.03668	0.93 (0.90–0.95)	<5.048	81.66% (78.10–84.87)	86.67% (75.41–94.06)	<0.0001
CEP68	1.610 ± 0.08263	0.6641 ± 0.02243	0.92 (0.89–0.95)	<1.113	81.66% (78.10–84.87)	83.33% (71.48–91.71)	<0.0001
HADH	1.056 ± 0.1059	−0.08562 ± 0.02584	0.92 (0.87–0.95)	<0.4448	83.74% (80.32–86.79)	90.00% (79.49–96.24)	<0.0001
ADAM33	0.8487 ± 0.08717	−0.01361 ± 0.01299	0.92 (0.87–0.97)	<0.3385	88.28% (85.23–90.90)	88.33% (77.43–95.18)	<0.0001
EPN3	0.5513 ± 0.1724	2.411 ± 0.04022	0.92 (0.89–0.95)	>1.596	82.04% (78.50–85.22)	90.00% (79.49–96.24)	<0.0001
ZNF8	−0.6821 ± 0.06841	0.1177 ± 0.02107	0.92 (0.88–0.96)	>−0.3715	84.69% (81.33–87.65)	88.33% (77.43–95.18)	<0.0001
DTWD1	0.1174 ± 0.07557	−0.7459 ± 0.02202	0.92 (0.89–0.95)	<−0.3011	84.12% (80.72–87.13)	90.00% (79.49–96.24)	<0.0001
PYGM	1.132 ± 0.1535	−0.3815 ± 0.02526	0.92 (0.88–0.96)	<0.09362	85.07% (81.74–88.00)	83.33% (71.48–91.71)	<0.0001
TDRD10	1.561 ± 0.1077	0.4343 ± 0.01890	0.91 (0.87–0.96)	<0.8479	84.88% (81.54–87.82)	86.67% (75.41–94.06)	<0.0001
SPINT2	0.9341 ± 0.1939	2.351 ± 0.02249	0.90 (0.87–0.93)	>1.981	81.85% (78.30–85.05)	83.33% (71.48–91.71)	<0.0001

**Table 3:** Comparison of expression patterns of the selected 20 DE mRNAs with the results of this study and other tumor studies.

Symbol	Description	NCBI Gene ID	Expression Pattern in This Study	Expression Patterns in Other Studies Related to Breast Cancer	Expression Patterns in Different Types of Cancer
ING3	Inhibitor Of Growth Family Member 3	54556	Downregulation	Downregulation [33,34]	Downregulated in liver cancer, head and neck cancer and colorectal cancer [35,36,37]
SNF1LK2	Salt Inducible Kinase 2	23235	Downregulation	Downregulation [38,39,40]	Downregulated in gastric cancer [41]
HBA2	Hemoglobin Subunit Alpha 2	3040	Downregulation	-	-
KCNIP2	Potassium Voltage-Gated Channel Interacting Protein 2	30819	Downregulation	-	-
SYF2	SYF2 Pre-MRNA Splicing Factor	25949	Downregulation	Upregulation [23]	Upregulated in epithelial ovarian cancer [22]
CPN2	Carboxypeptidase N Subunit 2	1370	Downregulation	Upregulation [25]	Upregulated in lung cancer [24]
EPB42	Erythrocyte Membrane Protein Band 4.2	2038	Downregulation	-	Downregulated in pancreatic cancer [42]
EMCN	Endomucin	51705	Downregulation	Downregulation [43]	Downregulated in renal cancer [44]
CEP68	Centrosomal Protein 68	23177	Downregulation	-	-
HADH	Hydroxyacyl-CoA Dehydrogenase	3033	Downregulation	-	Downregulated in renal cancer and gastric cancer [45,46,47]
ADAM33	ADAM Metallopeptidase Domain 33	80332	Downregulation	Downregulation [48,49]	
DTWD1	DTW Domain Containing 1	56986	Downregulation	-	Downregulated in gastric cancer [50]
PYGM	Glycogen Phosphorylase, Muscle Associated	5837	Downregulation	-	Downregulated in head and neck cancer [51]
TDRD10	Tudor Domain Containing 10	126668	Downregulation	Downregulation [52]	-
AP1M2	Adaptor Related Protein Complex 1 Subunit Mu 2	10053	Upregulation	-	-
EVPL	Envoplakin	2125	Upregulation	-	-
TOMM40	Translocase Of Outer Mitochondrial Membrane 40	10452	Upregulation	-	-
EPN3	Epsin 3	55040	Upregulation	Upregulation [28]	Downregulated in gastric cancer [26] Upregulated in glioblastoma [27]
ZNF8	Zinc Finger Protein 8	7554	Upregulation		
SPINT2	Serine Peptidase Inhibitor, Kunitz Type 2	10653	Upregulation	Upregulation [32] Downregulation [29]	Downregulated in liver, renal, gastric, cervical, prostate cancer and medulloblastoma [29,30,31]

## Data Availability

The data presented in this study are publicly available in the TCGA database, and the analysis and results presented in this work do not infringe copyright.
